# Seizure phenotypes and short-term prognosis in neonatal lupus: a multicenter retrospective cohort in China

**DOI:** 10.3389/fimmu.2026.1721089

**Published:** 2026-02-05

**Authors:** Wenqiang Sun, Xinyun Jin, Yihui Li, Xue Liu, Linzhou Zhu, Minqian Zhou, Zhirong Xie, Lili Li, Yanliang Yu, Yue Jiang, Jinhui Hu, Jie Huo, Huawei Wang, Haifeng Geng, Wenmei Li, Mengzhao Li, Yuanyuan Peng, Xihui Zhou, Xueping Zhu

**Affiliations:** 1 Department of Neonatology, Children’s Hospital of Soochow University, Suzhou, China; 2 Suzhou Municipal Key Laboratory of Neonatal Major Disease Rescue and Early-Life Prevention of Adult Chronic Diseases, Suzhou, China; 3 Department of Infectious Diseases, Soochow University Affiliated Children’s Hospital, Suzhou, China; 4 School of Public Health, Suzhou Medical College of Soochow University, Suzhou, China; 5 Department of Neonatology, The Affiliated Suzhou Hospital of Nanjing Medical University, Suzhou, China; 6 Department of Neonatology, Shenzhen Maternity and Child Healthcare Hospital, The First School of Clinical Medicine, Southern Medical University, Shenzhen, China; 7 Department of Neonatology, The Affiliated Yantai Yuhuangding Hospital of Qingdao University, Yantai, China; 8 Neonatal Medical Center, Huai’ an Maternity and Child Health Care Hospital, Huaian, China; 9 Department of Neonatology, Yangzhou Maternity and Child Health Care Hospital, Yangzhou, China; 10 Department of Child and Adolescent Healthcare, Children’s Hospital of Soochow University, Suzhou, China; 11 Department of Nephrology and Immunology, Children’s Hospital of Soochow University, Suzhou, China; 12 Department of Pediatrics, The First Affiliated Hospital of Xi’an Jiaotong University, Xi’an, China

**Keywords:** autoantibodies, clinical characteristics, neonatal lupus, neurodevelopmental outcomes, seizures

## Abstract

**Objectives:**

Neonatal lupus erythematosus (NLE) is an antibody-mediated autoimmune disorder that can affect neurologic outcomes. Seizures are an uncommon but clinically important phenotype. This study assessed clinical features, neuroimaging findings, and short-term outcomes in infants with NLE and seizures.

**Methods:**

We conducted a multicenter retrospective cohort study of infants with NLE admitted to seven tertiary centers in China. Infants were categorized as epileptic seizures (ES), non-epileptic CNS involvement (NE), or no CNS involvement (nCNS). Maternal characteristics and medications during pregnancy, infant clinical manifestations, laboratory indices, autoantibodies, EEG findings, and neuroimaging were extracted from medical records. Group comparisons and multivariable logistic regression were performed to identify factors associated with ES. Follow-up was conducted to 6 months of age.

**Results:**

Among 246 infants with NLE, 17 (6.91%) had seizures, 39 (15.85%) had non-seizure CNS involvement, and 190 (77.24%) had no CNS involvement. All ES infants presented with acute symptomatic seizures, predominantly focal. Neuroimaging abnormalities were common, most frequently intracranial hemorrhage. Anti-SSA/Ro positivity was universal in ES cases and significantly higher than in nCNS (*P* < 0.05). Maternal hydroxychloroquine (HCQ) use was less frequent in ES than nCNS (*P* < 0.05). Regarding organ involvement, the ES group showed higher rates of thrombocytopenia, coagulation abnormalities, hypocomplementemia, and pancytopenia than the nCNS group, as well as higher frequencies of hypocomplementemia and pancytopenia compared with the NE group (all *P* < 0.05). Multivariate regression analysis revealed that maternal HCQ use during pregnancy was independently associated with a lower odds of ES (OR: 0.066, 95% CI: 0.015–0.288, *P* = 0.001). No recurrent seizures were observed in neonates in the ES group after hospital discharge. However, nine infants exhibited varying degrees of developmental delay.

**Conclusions:**

NLE infants with seizures often exhibit focal seizures and structural brain injury, associated with anti-SSA/Ro positivity, limited maternal HCQ exposure, and hematologic abnormalities. Despite good seizure control, developmental delay was frequent, indicating risk for adverse neurodevelopment.

## Introduction

Neonatal lupus erythematosus (NLE) is a passively acquired autoimmune disease mediated by the transplacental transfer of maternal autoantibodies, primarily anti-SSA/Ro and anti-SSB/La. The disorder can involve multiple organ systems, with typical manifestations including annular cutaneous rash, congenital atrioventricular block, cytopenias, and hepatic dysfunction (1–3). Historically, clinical attention has focused on cardiac and cutaneous involvement, particularly congenital heart block, the most severe complication, which results from maternal autoantibody–induced inflammatory and fibrotic changes in the fetal myocardium (2, 4).

However, accumulating evidence suggests that NLE can also affect the central nervous system (CNS), and neurologic involvement is emerging as a clinically relevant concern. Maternal anti-Ro/La antibodies may impair neurodevelopment through immune-mediated mechanisms and, during the period of immature blood–brain barrier function, contribute to serious neurologic injury such as seizures, hydrocephalus, intracranial hemorrhage, and white matter damage; Recent follow-up studies further indicate an increased long-term risk of neurocognitive delay, attention-deficit/hyperactivity disorder (ADHD), and related developmental abnormalities in affected infants (5–8).

Notably, seizures represent one of the most acute and severe neurologic manifestations of NLE. They not only endanger neonatal vital stability but may also exert lasting adverse effects on long-term neurocognitive development (9, 10). Although several studies have described neurologic involvement in NLE, most are limited to case reports, and investigations specifically addressing NLE with seizures remain scarce (8). Moreover, systematic analyses incorporating maternal factors, immunologic markers, neurophysiology, and neuroimaging are lacking. This knowledge gap hinders early identification and timely intervention for these high-risk infants. Therefore, delineating the clinical characteristics of NLE with seizures has become an urgent clinical priority.

Therefore, this multicenter, large-sample study aimed to systematically characterize the clinical features and short-term outcomes of neonates with NLE and seizures. By exploring the associations between maternal factors, autoantibody profiles, and neurologic assessments with clinical manifestations, we sought to provide evidence to aid clinicians in recognizing and managing these high-risk infants, while also laying a foundation for future mechanistic studies and individualized management strategies.

## Materials and methods

### Study design

This was a multicenter retrospective clinical study. Infants diagnosed with NLE and admitted between January 1, 2011, and December 31, 2023, were identified from seven tertiary centers in China: Children’s Hospital of Soochow University, the First Affiliated Hospital of Xi’an Jiaotong University, the Affiliated Suzhou Hospital of Nanjing Medical University, Shenzhen Maternity and Child Healthcare Hospital, the Affiliated Yantai Yuhuangding Hospital of Qingdao University, Huai’an Maternal and Child Health Hospital, and Yangzhou Maternity and Child Health Care Hospital. The diagnosis of NLE was established based on the presence of NLE-related autoantibodies together with compatible clinical manifestations (2, 11). All diagnoses were jointly confirmed by neonatologists and pediatric rheumatologists. Exclusion criteria included incomplete clinical data that could bias study results, seizures unrelated to NLE, and confirmed cases of genetic or metabolic disorders.

Patients were stratified into three groups according to the presence or absence of seizures and CNS involvement: epileptic seizures (ES), non-epileptic CNS involvement (NE), and no CNS involvement (nCNS). Clinical features were then compared across the three groups. The ES group was defined by electroclinical seizures confirmed by routine, video, or continuous electroencephalographic (EEG) monitoring and classified according to the International League Against Epilepsy (ILAE) neonatal seizure classification (12). When continuous EEG was unavailable, infants with recurrent clinically suspected seizures supported by EEG findings were also included. To exclude seizures unrelated to NLE, differential diagnoses were made based on perinatal history and standard evaluations, including hypoxic–ischemic encephalopathy, infection/meningitis, hypoglycemia, and electrolyte disturbances; cases with confirmed genetic or metabolic etiologies were excluded. NE was defined as central nervous system involvement on neuroimaging during hospitalization without clinical seizures and without electrographic seizures on EEG. Imaging abnormalities were uniformly adjudicated based on institutional radiology reports and classified in conjunction with the clinical course.

### Study protocol and ethics approval

This study is a retrospective analysis approved by the institutional ethics committee of the hospital (Ethics No. 2025CS253). Written informed consent was obtained from the patients or their legal guardians. The study conforms to the Code of Ethics of the World Medical Association (Declaration of Helsinki).

### Data acquisition and follow-up

Data were collected through systematic review of inpatient and outpatient electronic medical records, supplemented by telephone follow-up. Information was obtained at both the maternal and infant levels. Maternal data included history of underlying rheumatic disease [e.g., systemic lupus erythematosus (SLE), Sjogren’s syndrome (SS), mixed connective tissue disease (MCTD), photosensitivity symptoms (PS), isolated autoantibody positivity without overt disease (IAPWD)], medication use during pregnancy [glucocorticoids, hydroxychloroquine (HCQ), low-molecular-weight heparin (LMWH), aspirin, and immunosuppressants], and autoantibody testing (anti-SSA/Ro, anti-SSB/La, anti-U1-RNP, and others).

Infant data included demographic characteristics (sex, gestational age, birth weight), clinical manifestations (seizure occurrence and type, involvement of other organ systems), laboratory findings (complete blood count, coagulation profile, complement levels, and biochemical parameters), immunologic assays (autoantibody profile), and neuroimaging results. Neuroimaging assessments comprised cranial ultrasound, CT, and MRI, with particular attention to intracranial hemorrhage, hydrocephalus, and white matter injury. Cranial ultrasonography was the primary modality for neuroimaging screening in hospitalized neonates, with CT or MRI performed when abnormalities were detected, seizures occurred, or further assessment of structural brain injury was clinically indicated. Neurophysiologic evaluation included routine EEG, video/continuous EEG monitoring, and characterization of seizure-related electrographic features. In a subset of infants, neurodevelopmental assessment was performed using the *Bayley Scales of Infant Development*. Follow-up was conducted in outpatient clinics by neonatologists and pediatric developmental specialists, focusing on seizure recurrence, repeat EEG findings, and neurodevelopmental outcomes. All infants were followed until 6 months of age.

### Statistical analysis

Descriptive statistics were applied as follows: normally distributed continuous variables were expressed as mean ± standard deviation (SD), non-normally distributed continuous variables as median (P25, P75), and categorical variables as frequency and percentage. We compared ES *vs* NE (*P1*) and ES *vs* nCNS (*P2*) throughout the analyses. Continuous variables were compared using the independent-samples t test when normally distributed and the Mann–Whitney U test otherwise. Categorical variables were compared using the chi-square test (with continuity correction when appropriate) or Fisher’s exact test. We fitted a Firth-penalized logistic regression model comparing ES and nCNS, with gestational age forcibly included for baseline adjustment. Additional covariates were selected based on significant associations in univariate analyses, while near-invariant variables were excluded and collinearity was minimized. All statistical analyses were performed using R software (version 4.2.1). A two-sided *P* value < 0.05 was considered statistically significant.

## Results

### Patient selection

**Figure 1:**
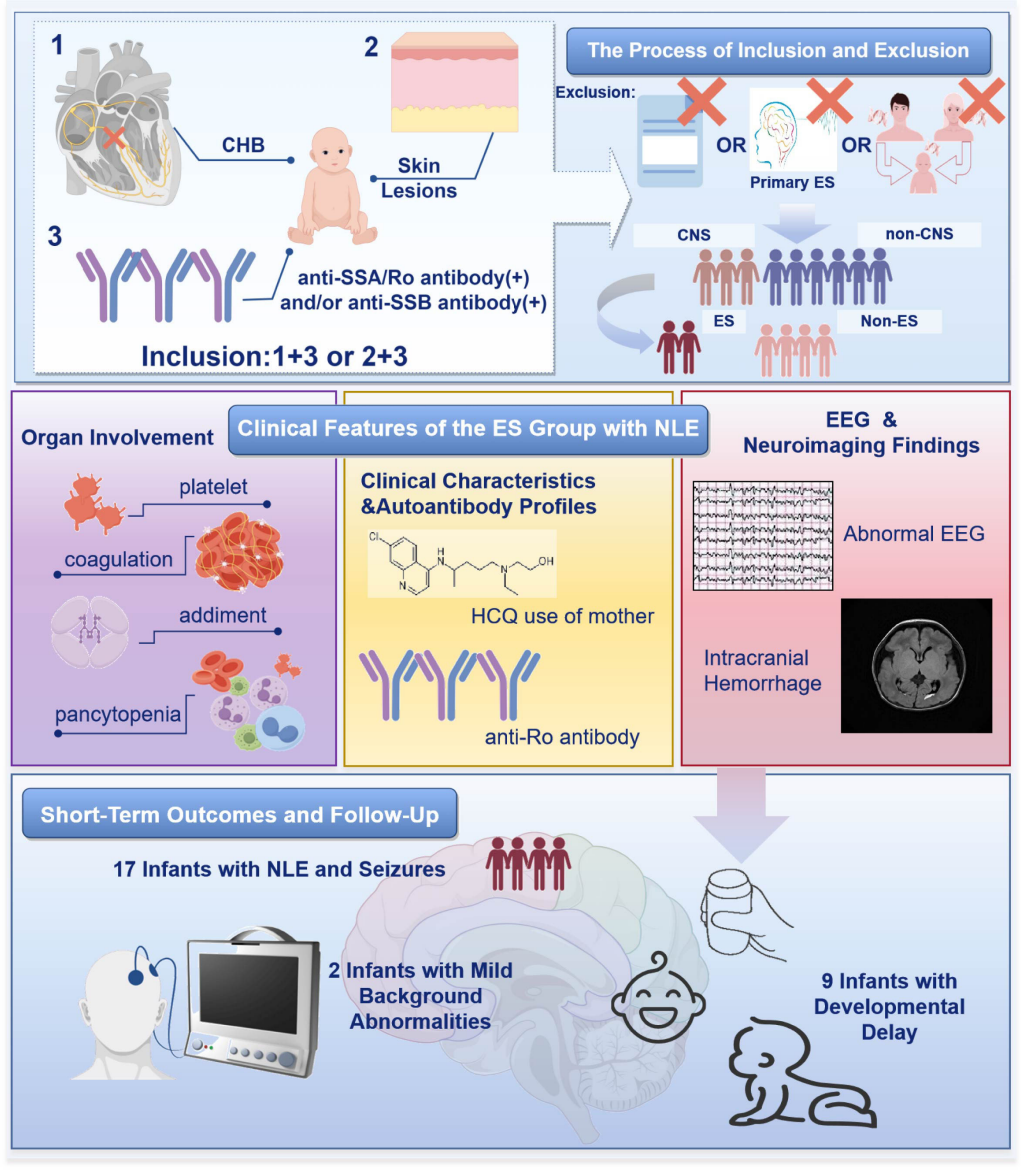
The flowchart of study. NLE, Neonatal Lupus Erythematosus; CHB, congenital heart block; CNS, Central Nervous System; ES, Epileptic Seizures (group with seizures); NE, Non-Epileptic CNS Involvement (group with CNS but no seizures); nCNS, No CNS Involvement (group without CNS involvement); EEG, electroencephalography.

The study flowchart is detailed in **Figure 1**. Between January 1, 2011, and December 31, 2023, a total of 267 infants with NLE were admitted. Nineteen were excluded due to incomplete clinical data or refusal of participation, and 2 due to confirmed genetic or metabolic disorders, leaving 246 infants eligible for analysis.

Of these, 120 were male and 126 female. The median gestational age was 36 + 2 (33 + 4, 38 + 1) weeks, and the median birth weight was 2,575.00 g (1,860.00, 3,050.00). Among the 246 infants, 190 (77.24%) had no CNS involvement, while 56 (22.76%) showed CNS manifestations. Seizures were present in 17 (6.91%), and 39 (15.85%) had non-epileptic CNS involvement.

### Clinical features, EEG, and neuroimaging findings of NLE infants with seizures

Clinical features, EEG, and neuroimaging findings of NLE infants with seizures see **Table 1**. Among the 17 infants with NLE who developed seizures, clinical analysis revealed the following major findings: females predominated (9/17, 52.94%), with a median gestational age of 36 + 1 weeks (33 + 5, 37 + 2) and a median birth weight of 2,440 g (1,880, 2,780). All infants presented with acute symptomatic seizures, most commonly focal seizures (12/17, 70.59%), followed by myoclonic seizures (4/17, 23.53%) and one case of generalized seizure (5.88%).

**Table 1:** Clinical features, EEG, and neuroimaging findings of NLE infants with seizures.

No.	Seizure characteristics	EEG	Neuroimaging	ASMs	Medication	Duration
1	focal seizures	abnormal background activity	PV-IVH, periventricular white matter hypodensity	+	PB	4 days
2	focal seizures	focal discharges	enlarged extracerebral spaces, cerebral edema, PV-IVH	–		
3	focal seizures	focal discharges	–	–		
4	myoclonic seizures	paroxysmal high-frequency low-amplitude oscillations	enlarged extracerebral spaces, hydrocephalus, SAH	+	DZP → PB	2 doses→ 6 days
5	focal seizures	focal discharges	–	–		
6	generalized seizures	generalized synchronous discharge	hydrocephalus, SEH, periventricular white matter hypodensity	+	DZP → PB	1doses→ 5 days
7	focal seizures	focal spike waves	SAH	–		
8	focal seizures	abnormal background activity	enlarged extracerebral spaces	–		
9	focal seizures	focal discharges	enlarged extracerebral spaces, SAH	+	PB	3 days
10	myoclonic seizures	abnormal background activity	PV-IVH, cerebral edema	+	PB	4 days
11	focal seizures	focal discharges	–	–		
12	focal seizures	focal discharges	SEH	+	PB	3 days
13	myoclonic seizures	abnormal background activity	hydrocephalus, PVL-IVH, enlarged extracerebral spaces	–		
14	focal seizures	focal discharges	hydrocephalus, SEH	–		
15	focal seizures	focal spike waves	hydrocephalus, SAH	+	DZP → PB	1 doses→ 5 days
16	myoclonic seizures	transient high-amplitude sharp waves	–	–		
17	focal seizures	focal discharges	SAH、cerebral edema	+	PB	3 days

EEG, Electroencephalography; NLE, Neonatal lupus erythematosus; ASMs, Anti-seizure Medication; PV-IVH, Periventricular–Intraventricular Hemorrhage; PB, Phenobarbital; SAH, subarachnoid hemorrhage; DZP, Diazepam; SEH, subependymal haemorrhage.

Electroencephalography demonstrated abnormalities in all patients. Focal epileptiform discharges were observed in 10 cases (58.82%), abnormal background activity in 4 (23.53%), and paroxysmal low-amplitude fast activity, generalized synchronous discharges, and transient high-amplitude sharp waves in one case each (5.88%).

Neuroimaging identified structural brain injury in 13 infants (76.47%). Intracranial hemorrhage was the most frequent finding (12/17, 70.59%), including subarachnoid hemorrhage (SAH) (5/17, 29.41%), periventricular–intraventricular hemorrhage (PV-IVH) (4/17, 23.53%), and subependymal hemorrhage (SEH) (3/17, 17.65%). Additional findings included enlarged extracerebral spaces (5/17, 29.41%), hydrocephalus (5/17, 29.41%), cerebral edema (2/17, 11.76%), and white matter injury (2/17, 11.76%). Eight infants (47.06%) required antiepileptic treatment during hospitalization for recurrent seizures, with phenobarbital administered in all cases and diazepam used in three during the acute phase.

### Baseline clinical characteristics and autoantibody profiles

Baseline clinical characteristics of infants and mothers see **Table 2**. Among the 246 mothers of infants with NLE, SLE was the most common underlying condition (136/246, 55.28%), followed by SS (51/246, 20.73%), PS (20/246, 8.13%), IAPWD (23/246, 9.35%), and MCTD (10/246, 4.07%). Seven mothers (2.85%) denied a history of autoimmune disease. During pregnancy, 223 mothers (90.65%) received glucocorticoids, 184 (74.80%) HCQ, 119 (48.37%) LMWH, 27 (10.98%) aspirin, and 15 (6.10%) immunosuppressants. Serological testing revealed that 193 mothers (78.46%) were positive for anti-SSA/Ro antibodies, 123 (50.00%) for anti-SSB/La, and 78 (31.71%) for anti-U1-RNP. Dual anti-Ro/La positivity was present in 109 cases (44.31%).

**Table 2:** Baseline clinical characteristics of infants and mothers.

Characteristics	ES group (n=17)	NE group(n=39)	nCNS group (n=190)	*P1*	*P2*
Male	8 (47.06%)	20 (51.28%)	92 (48.42%)	0.771	0.914
Gestational age	36 + 1(33 + 5, 37 + 2)	36 + 4(33 + 2, 39 + 0)	36 + 6(33 + 1, 39 + 0)	0.187	0.165
Birth weight	2440.00 (1880.00, 2780.00)	2450.00 (1850.00, 2950.00)	2575.00 (1860.00, 3150.00)	0.775	0.278
Maternal underlying diseases					
Systemic lupus erythematosus	10 (58.82%)	21 (53.84%)	105 (55.26%)	0.730	0.777
Sjögren’s syndrome	5 (29.41%)	8 (20.51%)	38 (20.00%)	0.468	0.359
MCTD	1 (5.88%)	2 (5.13%)	7 (3.68%)	1.000	1.000
Photosensitivity symptoms	0	5 (12.82%)	15 (7.89%)	0.309	0.618
IAPWD	1 (5.88%)	3 (7.69%)	19 (10.00%)	1.000	0.903
Denying autoimmune disease	0	1 (2.56%)	6 (3.16%)	1.000	1.000
Maternal use of anti-rheumatic drugs during pregnancy					
Glucocorticoids	15 (88.24%)	32 (82.05%)	176 (92.63%)	0.854	0.860
HCQ	9 (52.94%)	26 (66.67%)	149 (78.42%)	0.329	0.018
LMWH	9 (52.94%)	18 (46.15%)	92 (48.42)	0.640	0.721
Aspirin	2 (11.76%)	5 (12.82%)	20 (10.53%)	1.000	1.000
Immunosuppressants	1 (5.88%)	3 (7.69%)	11 (5.79%)	1.000	1.000

NLE, Neonatal lupus erythematosus; ES, Epileptic Seizures (group with seizures); NE, Non-Epileptic CNS Involvement (group with CNS but no seizures); nCNS, No CNS Involvement (group without CNS involvement); MCTD, Mixed Connective Tissue Disease; IAPWD, Isolated Autoantibody Positivity Without Overt Disease; HCQ, hydroxychloroquine; LMWH, low-molecular-weight heparin. *P1* represents the comparison between the ES group and the NE group; *P2* represents the comparison between the ES group and the nCNS group.

In the ES group (n = 17), 8 infants (47.06%) were male. The predominant maternal condition was SLE (10/17, 58.82%), followed by SS (5/17, 29.41%); MCTD and IAPWD were each observed in one case (5.88%). Regarding maternal treatment during pregnancy, glucocorticoids were administered in 15 cases (88.24%), HCQ in 9 (52.94%), LMWH in 9 (52.94%), aspirin in 2 (11.76%), and immunosuppressants in 1 (5.88%). Serological testing showed that all mothers of ES infants were positive for anti-SSA/Ro antibodies (17/17, 100.00%). Anti-SSB/La antibodies were detected in 8 cases (47.06%), anti-U1-RNP in 4 (23.53%), and dual anti-Ro/La positivity in 8 (47.06%).

Comparison of infants across the ES, NE, and nCNS groups showed no significant differences in sex distribution, gestational age, birth weight, or types of maternal autoimmune disease (all *P* > 0.05).

Regarding maternal treatment, the rate of HCQ use during pregnancy was significantly lower in the ES group (9/17, 52.94%) compared with the nCNS group (149/190, 78.42%; *P* = 0.018), whereas the use of glucocorticoids, LMWH, aspirin, and immunosuppressants did not differ significantly among the three groups (all *P* > 0.05).

Autoantibody profiles of infants see **Table 3**. For serologic markers, the prevalence of anti-Ro antibody positivity was significantly higher in the ES group (17/17, 100.00%) than in the nCNS group (149/190, 78.42%; *P* = 0.032), but was not significantly different from the NE group (*P* > 0.05). No significant intergroup differences were observed for anti-SSB/La, anti-U1-RNP, or dual anti-Ro/La antibody positivity (all *P* > 0.05).

**Table 3:** Expression of autoimmune antibodies in infants with NLE.

Characteristics	ES group (n=17)	NE group (n=39)	nCNS group (n=190)	*P1*	*P2*
Anti-SSA/Ro	17 (100.00%)	33 (84.62%)	149 (78.42%)	0.163	0.032
Anti-SSB/La	8 (47.06%)	19 (48.72%)	96 (50.53%)	1.000	0.784
Anti-U1-RNP	4 (23.53%)	10 (25.64%)	64 (33.68%)	1.000	0.559
Dual anti-Ro/La positivity	8 (47.06%)	16 (41.03%)	85 (44.74%)	0.675	0.854

NLE, Neonatal lupus erythematosus; ES, Epileptic Seizures (group with seizures); NE, Non-Epileptic CNS Involvement (group with CNS but no seizures); nCNS, No CNS Involvement (group without CNS involvement).

### Organ involvement in infants with NLE

Organ involvement in infants with NLE see **Table 4**. Among the 246 infants with NLE, organ involvement most frequently affected cutaneous (208/246, 84.55%), followed by gastrointestinal (163/246, 66.26%), hematological (145/246, 58.94%), hypocomplementemia (88/246, 35.77%) and CHB (57/246, 23.17%). Within hematologic abnormalities, anemia was most common (106/246, 43.10%), followed by thrombocytopenia (90/246, 36.58%), neutropenia/deficiency (80/246, 32.52%), pancytopenia (43/246, 17.48%), and coagulation abnormalities (36/246, 14.63%).

**Table 4:** Organ involvement in infants with NLE.

Organ involvement in NLE	ES group (n=17)	NE group(n=39)	nCNS group (n=190)	*P1*	*P2*
Cutaneous (rash)	15 (88.24%)	32 (82.05%)	161 (84.73%)	0.854	0.699
Hematological					
Total	12 (70.59%)	24 (61.54%)	109 (57.37%)	0.516	0.289
Anemia	10 (58.82%)	17 (43.59%)	79 (41.58%)	0.294	0.169
Neutropenia/deficiency	7 (41.18%)	12 (30.77%)	61 (32.11%)	0.449	0.445
Thrombocytopenia	10 (58.82%)	14 (35.90%)	66 (34.74%)	0.111	0.048
Coagulation abnormalities	5 (29.41%)	9 (23.08%)	22 (11.58%)	0.615	0.036
Pancytopenia	6 (35.29%)	8 (20.51%)	29 (15.26%)	0.036	0.035
Hypocomplementemia	11 (64.71%)	12 (30.77%)	65 (34.21%)	0.018	0.012
Congenital heart block	5 (29.41%)	11 (28.21%)	41 (21.58%)	0.927	0.457
Gastrointestinal	12 (70.59%)	26 (66.67%)	125 (65.79%)	0.773	0.689

NLE, Neonatal lupus erythematosus; ES, Epileptic Seizures (group with seizures); NE, Non-Epileptic CNS Involvement (group with CNS but no seizures); nCNS, No CNS Involvement (group without CNS involvement).

**Figure 2:**
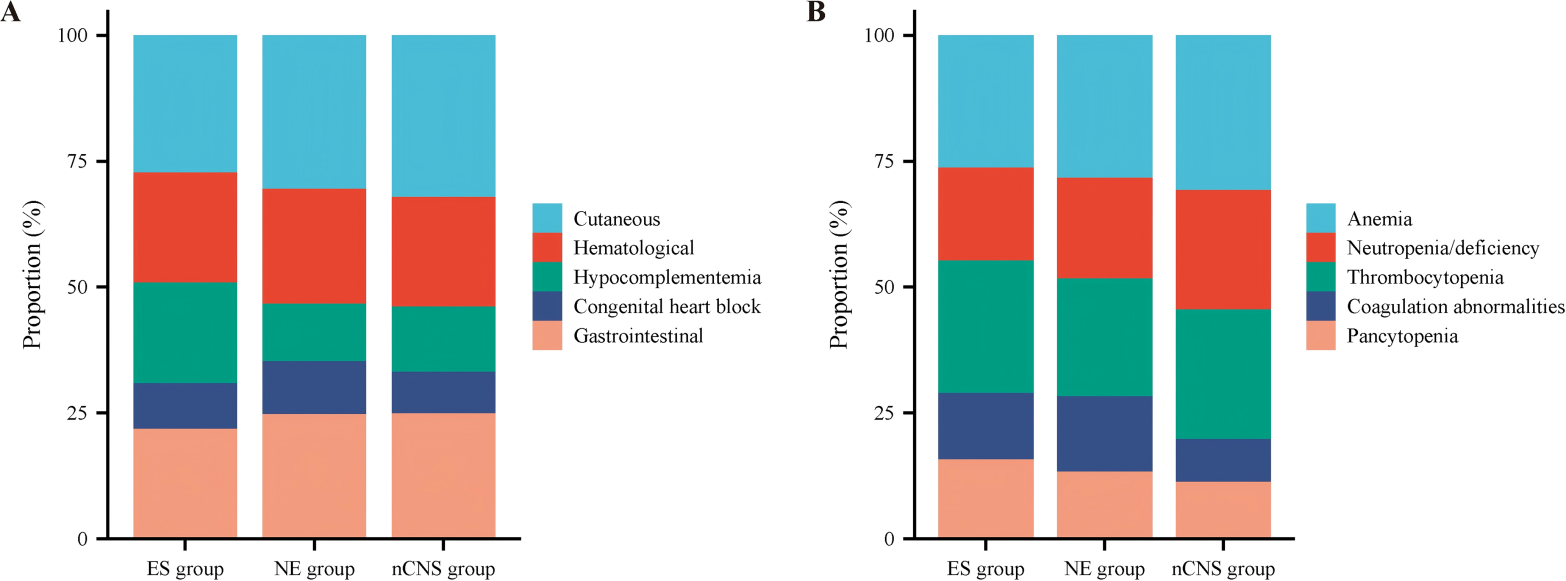
The distribution of organ involvement **(A)** and blood system involvement **(B)** in infants of NLE. NLE, Neonatal Lupus Erythematosus; ES, Epileptic Seizures (group with seizures); NE, Non-Epileptic CNS Involvement (group with CNS but no seizures); nCNS, No CNS Involvement (group without CNS involvement).

In the ES group (n = 17), the frequencies of organ involvement were, in descending order, cutaneous (15/17, 88.24%), hematological (12/17, 70.59%), gastrointestinal (12/17, 70.59%), hypocomplementemia (11/17, 64.71%), and congenital heart block (CHB) (5/17, 29.41%). Hematological involvement including anemia (10/17, 58.82%), thrombocytopenia (10/17, 58.82%), Neutropenia/deficiency (7/17, 41.18%), pancytopenia (6/17, 35.29%), and coagulation abnormalities (5/17, 29.41%). The distribution of organ involvement and blood system involvement is detailed in **Table 4** and **Figures 2A, B**. When comparing organ involvement across groups, no significant intergroup differences were found for cutaneous, gastrointestinal, or cardiac involvement (all *P* > 0.05). Compared with the nCNS group, the ES group had significantly higher rates of thrombocytopenia (58.82% *vs* 34.74%, *P* = 0.048), coagulation abnormalities (29.41% *vs* 11.58%, *P* = 0.036), hypocomplementemia (64.71% *vs* 34.21%, *P* = 0.012), and pancytopenia (35.29% *vs* 15.26%, *P* = 0.035). Compared with the NE group, hypocomplementemia (64.71% *vs* 30.77%, *P* = 0.018) and pancytopenia (35.29% *vs* 20.51%, *P* = 0.036) were also more frequent in the ES group. No significant differences were observed between the ES and nCNS groups for anemia or neutropenia/deficiency (all *P* > 0.05), or between the ES and NE groups for anemia, neutropenia/deficiency, thrombocytopenia, or coagulation abnormalities (all *P* > 0.05).

A multivariable logistic regression analysis was performed with ES as the outcome, comparing the ES and nCNS groups. Due to the limited number of ES events, Firth’s penalized logistic regression was applied. Gestational age was included *a priori* for adjustment, and additional covariates entered into the model were those showing significant associations in univariate analyses, including maternal HCQ use, thrombocytopenia, coagulation abnormalities, hypocomplementemia, and pancytopenia. Maternal HCQ use during pregnancy was independently associated with a lower odds of ES (OR: 0.066, 95% CI: 0.015–0.288, *P* = 0.001).

### Short-term outcomes and follow-up

During the period from hospital discharge to the 6-month follow-up, none of the 17 infants with NLE experienced recurrent seizures. Two infants did not undergo repeat EEG. Among the remaining 15, 13 (86.67%) had normal EEG findings, while 2 (cases 6 and 13) showed mild background abnormalities that were no longer evident on short-term EEG reevaluation at 6 months. Notably, 9 infants (52.94%) demonstrated varying degrees of developmental delay.

## Discussion

The neonatal nervous system is immature, characterized by heightened neuronal excitability and underdeveloped inhibitory mechanisms, which increase susceptibility to seizures (13, 14). In our cohort of 246 infants with NLE, approximately one-quarter demonstrated CNS involvement, with nearly 7% experiencing acute symptomatic seizures. Among affected infants, focal seizures predominated, and more than two-thirds exhibited neuroimaging abnormalities, most commonly intracranial hemorrhage. EEG abnormalities were observed in all infants, underscoring the high sensitivity of EEG monitoring in identifying neurologic risk in NLE.

Notably, the prevalence of anti-SSA/Ro antibody positivity was 100% in the ES group, which was significantly higher than that in the nCNS group. This finding suggests an association between anti-SSA/Ro status and the occurrence of seizures in infants with NLE, indicating that anti-SSA/Ro antibodies may serve as an immunological marker of increased neurological risk. However, this association may also be influenced by other perinatal confounding factors. From a mechanistic perspective, previous studies have shown that anti-SSA/Ro antibodies can bind to and inhibit voltage-gated calcium channels in cardiomyocytes (15). Given the pivotal role of these channels in neuronal excitability and synaptic transmission (16), it is plausible that such antibodies may lower the seizure threshold by modulating calcium influx. Furthermore, the detection of anti-SSA/Ro antibodies and evidence of localized immune responses in the cerebrospinal fluid of patients with primary SS with CNS involvement (17) further support the hypothesis of an immune-mediated mechanism in seizure pathogenesis. However, direct experimental evidence demonstrating pathogenic effects of these antibodies on neuronal membranes remains lacking, warranting further mechanistic investigation. In terms of maternal treatment, HCQ use during pregnancy was less frequent in the ES group than in the nCNS group, suggesting an association between maternal HCQ exposure and seizure occurrence in infants with NLE. This observation is consistent with prior studies reporting an association between HCQ exposure and reduced cardiac and cutaneous manifestations in NLE (18, 19).

Unlike previous reports that have primarily emphasized cutaneous, cardiac, and hepatic involvement in NLE (1, 2), our study demonstrated that hematological abnormalities were more prominent in the ES group compared with the other groups. These findings suggest that hematologic abnormalities may play an important role in the development of CNS injury in NLE. Thrombocytopenia and coagulation dysfunction may increase the risk of intracranial hemorrhage (20, 21). In addition, platelet activation by autoantibodies may contribute to immune dysregulation through direct interactions with immune cells and could synergize with IgG to promote Interferon-alpha production. Hypocomplementemia, in turn, reflects immune complex–mediated inflammation and complement activation (6, 22). Together, these mechanisms may exacerbate the inherent fragility of fetal cerebral vasculature, thereby increasing susceptibility to brain injury and seizures. Compared with the NE group, the more severe hematologic abnormalities observed in the ES group further support a potential link between hematologic involvement and neurologic manifestations. Clinically, these results highlight the importance of close hematologic monitoring and early intervention in NLE, particularly in infants presenting with seizures. Prompt correction of thrombocytopenia or coagulopathy may help reduce the risk of intracranial hemorrhage and secondary seizures.

During the follow-up period from hospital discharge to 6 months of age, none of the infants in the ES group experienced recurrent seizures. Most demonstrated normalization of EEG findings, indicating that acute symptomatic seizures can be well controlled in the short term when promptly recognized and managed. However, more than half of the infants exhibited varying degrees of developmental delay, highlighting that seizure control does not equate to elimination of neurodevelopmental risk. Based on the neuroimaging and hematologic data from our cohort, a plausible explanation is that maternal anti-Ro/SSA antibodies, transferred across the placenta, may trigger complement-mediated inflammatory responses in the fetus. When compounded by early hematologic abnormalities such as thrombocytopenia and hypocomplementemia, these processes may increase cerebrovascular fragility and the risk of intracranial hemorrhage or white matter injury. In addition, the immature blood–brain barrier and the perinatal inflammatory milieu may activate microglia and amplify cytokine cascades, leading to predominantly structural or network-level “subclinical” injuries (23, 24). Such mechanisms may continue to impair motor, language, and cognitive development even after apparent EEG recovery. These findings suggest that clinical management should evolve from a single focus on seizure control to a dual strategy of “seizure control plus developmental protection.” At the maternal level, immune status and treatment during pregnancy may modulate fetal inflammatory pathways (25, 26).

The major strength of this study is that it is based on a multicenter clinical cohort, enabling a systematic characterization of NLE infants with seizures across electrophysiologic, neuroimaging, and immunologic domains. However, several limitations should be acknowledged. As a retrospective multicenter study, the timing and duration of EEG monitoring, neuroimaging protocols, and the administration of Bayley scale assessments were not fully standardized across centers, and incomplete records were unavoidable. These factors may have introduced information and selection bias, thereby limiting the precision and generalizability of analyses related to neurodevelopmental outcomes and certain associations. In addition, the small number of cases in the ES group (n = 17) constrained statistical power. Although our findings suggest a potential neuroprotective effect of maternal immunotherapy, this observation warrants confirmation in prospective studies. Future multicenter investigations with standardized assessments, longer follow-up, and mechanistic exploration will be essential to optimize maternal–fetal management strategies and improve outcomes in affected infants.

## Conclusion

This study systematically characterized the clinical features and short-term outcomes of NLE infants with seizures. Our findings demonstrate that these infants frequently present with electrophysiologic and neuroimaging abnormalities and are strongly associated with maternal anti-SSA/Ro antibody positivity. In addition, maternal HCQ use was lower in the ES group, suggesting that immune-mediated inflammation and hematologic abnormalities may play important roles in pathogenesis. Although seizure control was favorable during short-term follow-up, more than half of the infants exhibited developmental delay, indicating that underlying neurologic injury may have lasting consequences. These results highlight that clinical management should extend beyond seizure control to include systematic neurodevelopmental follow-up and early intervention. Future multicenter studies with longer follow-up and mechanistic exploration will be needed to validate the potential protective effects of maternal immunotherapy and to optimize maternal–fetal management strategies.

## Data Availability

The original contributions presented in the study are included in the article/supplementary material. Further inquiries can be directed to the corresponding authors.
